# Key Technologies of Synthetic Biology in Industrial Microbiology

**DOI:** 10.3390/microorganisms13102343

**Published:** 2025-10-13

**Authors:** Xinyue Jiang, Jiayi Ji, Qi Yang, Yao Dou, Yujue Li, Xiaoyu Yang, Chunying Liu, Shaohua Dou, Liang Dong

**Affiliations:** 1 College of Life and Health, Dalian University, Dalian 116622, China; 13199062388@163.com (X.J.); m18246595901@163.com (J.J.); 15504325267@163.com (Q.Y.); 15601630413@163.com (Y.L.); 18742590471@163.com (X.Y.); lcycj321@163.com (C.L.); 2 College of Economics and Management, Huazhong Agricultural University, Wuhan 430070, China; angelyao@163.com

**Keywords:** industrial microbiology, synthetic biology, gene editing technology, metabolic engineering, high-throughput screening, synthetic genomics

## Abstract

Industrial microorganisms have a wide range of applications in biomanufacturing, energy production, environmental protectionpharmaceutical development, etc. Synthetic biology has revolutionised the field of industrial microorganisms by designing, constructing and optimising biological systems. The aim of this study is to discuss the key technologies of synthetic biology in industrial microorganisms and their application prospects. Gene editing technology, one of the core tools of synthetic biology, enables researchers to precisely modify microbial genomes to optimise their metabolic pathways or introduce new functions. Metabolic engineering, as an important direction for the application of synthetic biology in industrial microorganisms, enables the efficient synthesis of target products by optimising and reconstructing the metabolic pathways of microorganisms. The development of high-throughput screening and automated platforms has enabled large-scale gene editing and metabolic engineering experiments. The application of synthetic genomics promises to develop microbes with highly customised functions. However, there are still many challenges in this field, and future research still requires interdisciplinary collaboration to drive the application of synthetic biology in industrial microorganisms to new heights.

## 1. Introduction

**Figure 1:**
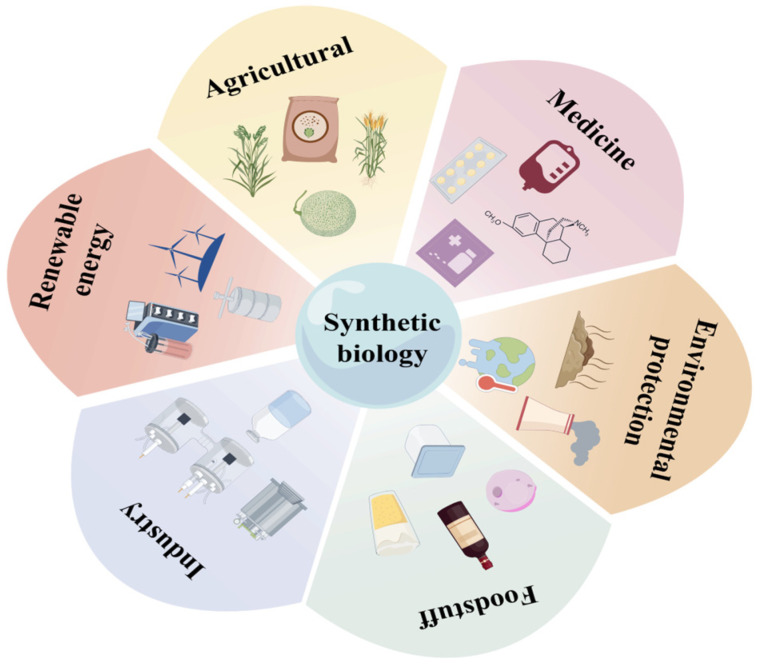
Applications of Synthetic Biology in Renewable Energy, Agriculture, Medicine, Environmental Protection, Food, and Industrial Sectors.

Synthetic biology is an emerging interdisciplinary field that integrates biochemistry, molecular biology, informatics, and statistics, among other disciplines. It employs synthetic techniques to enable the targeted design and modification of cells or organisms, thereby endowing them with novel and specialized functions [1]. This field facilitates the design and fabrication of new biological components, devices, and systems, ultimately contributing to the creation of “artificial life.” Additionally, it allows for the redesign and modification of existing natural biological systems, resulting in what is termed “engineered life” [2,3,4]. Key research directions in synthetic biology include: the design and construction of genetic regulatory networks to program and control cellular behaviors; the optimization and reconstruction of metabolic pathways to enhance the yield and efficiency of target products; the design and synthesis of minimal or functional genomes for constructing organisms with new capabilities; the engineering of proteins to confer novel functions or improve their performance; and the application of synthetic biology in modifying microbial cells for biomanufacturing and environmental remediation [5,6,7,8,9,10]. In summary, synthetic biology offers the potential to construct new biological systems that address pressing challenges such as environmental management. It also leverages the diversity of existing biological entities to create predictable and controllable synthetic assemblies tailored to human needs, including applications in pharmaceuticals, disease diagnosis, and therapy [11,12,13]. Figure 1 provides an overview of the application domains of synthetic biology.

### 1.1. Overall Synthetic Biology Market

Amid increasing public concern regarding health and environmental issues, market demand for synthetic biology continues to expand. According to the “China Synthetic Biology Industry Outlook and Investment Strategy Planning Analysis Report, 2025–2030” released by the China Business Industry Research Institute, the global synthetic biology market reached approximately $12.2 billion in 2022, reflecting a year-on-year growth of 28.42%. It is projected to grow to around $15.1 billion in 2023 and $19 billion in 2024. Analysts further estimate that the global market will reach $24.3 billion by 2025. In terms of application fields, healthcare and chemical/energy sectors dominate the market, accounting for 55.58% and 32.09% of the market share, respectively. Agricultural technology, food and beverage, and information technology represent smaller shares, at 6.98%, 4.83%, and 0.47%. Within secondary sectors, enterprises focusing on gene therapy, cell therapy, drug synthesis, and bio-based materials show relatively concentrated activity and have become recent hotspots for investment [14].

### 1.2. The Importance of Industrial Microorganisms

Industrial microorganisms encompass a variety of microbial species—including bacteria, yeasts, fungi, actinomycetes, and viruses—that are employed in industrial production processes. Owing to their distinctive metabolic characteristics and genetic adaptability, these microorganisms play an essential role in diverse sectors such as agriculture, food processing, pharmaceuticals, and even aspects of human health including psychological and reproductive well-being [15]. In traditional industries, microorganisms are harnessed to produce fermented foods such as yogurt, cheese, soy sauce, beer, and wine [16,17,18,19,20]. They are also pivotal in the synthesis of food additives, including vitamins, amino acids, pigments, and flavor compounds [21,22,23,24]. Furthermore, industrial microorganisms serve as a major source of enzymes, which are extensively applied in sectors such as food processing, detergent manufacturing, textiles, and paper production [25,26,27,28,29,30]. Additionally, many antibiotics—including *penicillin* and *streptomycin*—are derived from microbial biosynthesis [31,32,33]. Microorganisms are also employed in the production of organic acids such as citric acid, lactic acid, and acetic acid, as well as industrial solvents including ethanol and acetone [34,35,36,37,38]. In modern biotechnology, the application scope of industrial microorganisms has significantly expanded. In the field of biomanufacturing, microbial cell are now engineered for the efficient production of biofuels such as bioethanol, biodiesel, and fatty hydrocarbons [39], as well as bio-based chemicals including lactic acid [40] and C4 organic acids [41]. They are also harnessed to synthesize environmentally friendly biodegradable plastics, such as polylactic acid (PLA) [42] and polyhydroxyalkanoates (PHA) [43]. In pharmaceutical and high-value compound manufacturing, microorganisms are utilized to produce antibiotics [44,45], vaccines [46,47], and hormones [48,49], as well as complex natural products like terpenoids [50,51] and alkaloids [52]. Within environmental remediation, industrial microbes contribute to the degradation of pollutants such as petroleum hydrocarbons [53], plastics [54], and pesticides [55]. They are also applied in wastewater treatment to remove organic matter, nitrogen, and phosphorus [56,57]. Furthermore, microorganisms are employed in the cosmetics industry for the production of hyaluronic acid [58], ergothioneine [59], and yeast fermentation filtrates [60]. Table 1 provides a detailed overview of specific applications of industrial microorganisms. As biotechnology continues to advance, the potential of industrial microorganisms is expected to grow further, offering substantial support for economic, social, and environmental sustainability. In the future, driven by synthetic biology and artificial intelligence, industrial microorganisms are poised to become a central driving force in the emerging bioeconomy.

### 1.3. Significance of Integrating Synthetic Biology with Industrial Microbiology

The combination of synthetic biology and industrial microorganisms represents a revolutionary advancement in biotechnology [86]. By applying engineering principles and technical strategies from synthetic biology to industrial microorganisms, researchers can now design and construct microbial cell factories [87] with tailored functions, enabling groundbreaking innovations and applications across biomanufacturing, healthcare, energy, and environmental remediation. For example, Xu et al. [88] engineered *Corynebacterium glutamicum* by introducing exogenous fructokinase ScrK and ADP-dependent phosphofructokinase, while overexpressing the ATP synthase gene—which catalyzes the phosphorylation of ADP to ATP, thereby enhancing the intracellular ATP synthesis rate. The modified strain achieved an L-lysine yield of 221.30 g/L using fructose as the primary carbon source, demonstrating the potential of rationally designed microbial cell factories to drastically improve production efficiency. Through techniques such as gene editing, metabolic engineering, and genome design, the capabilities of industrial microorganisms can be substantially enhanced. These approaches enable precise design and controllable production, greatly expanding the functional scope of industrial microorganisms and allowing them to play critical roles in diverse sectors [89]. This study systematically examines how key synthetic biology technologies are driving innovation in industrial microorganisms and discusses future application prospects and challenges.

## 2. Key Technologies of Synthetic Biology in Industrial Microorganisms

### 2.1. Gene Editing Technologies

Gene editing technologies enable precise modifications of target genes, including knockouts, knock-ins, substitutions, and functionally relevant point mutations [90]. These techniques alter gene structure to modulate specific gene functions, thereby influencing cellular characteristics or phenotypic outcomes. Traditional gene editing relies on DNA homologous recombination (HR)-mediated gene targeting, which suffers from low efficiency and has limited its broad application [91]. In recent years, more efficient genome editing tools have been developed, such as zinc finger nucleases (ZFNs), transcription activator-like effector nucleases (TALENs), and CRISPR (clustered regularly interspaced short palindromic repeats)-based systems [92].

#### 2.1.1. ZFNs

Zinc finger nuclease (ZFN) technology, introduced in 1996, represents the first generation of programmable genome editing tools. It operates through the sequence-specific recognition of DNA by zinc finger proteins (ZFPs), which are fused to the FokI endonuclease domain to form a functional editing complex [93]. In recent years, advances in protein engineering have enabled the fusion of the FokI cleavage domain to both the amino and carboxyl termini of zinc finger proteins, thereby expanding ZFN’s targeting range. Additionally, strategies such as attenuating the cleavage activity of FokI have been employed to enhance its specificity [94]. In a study by Lessard S et al. [95] hematopoietic stem cells (HSCs) were genetically modified using mRNA encoding ZFNs. The designed ZFNs targeted and disrupted a regulatory GATA motif within the BCL11A erythroid enhancer, leading to reactivation of fetal hemoglobin (HbF) expression.

#### 2.1.2. TALENs

Similar to ZFNs, transcription activator-like effector nucleases (TALENs) are constructed by fusing a DNA-binding domain derived from transcription activator-like effectors (TALEs) to the catalytic domain of FokI endonuclease [96]. TALEs were originally identified in *Xanthomonas* species and feature a modular DNA-binding domain composed of tandem repeats, with each repeat binding to a single nucleotide [97]. Each repeat unit comprises 34 amino acids, among which the highly variable residues at positions 12 and 13—known as repeat variable diresidues (RVDs)—determine nucleotide specificity. Due to the relative ease of designing TALE DNA-binding domains compared to zinc finger arrays, TALENs offer broader targeting flexibility and have been widely adopted across various applications in the life sciences [98].

**Figure 2:**
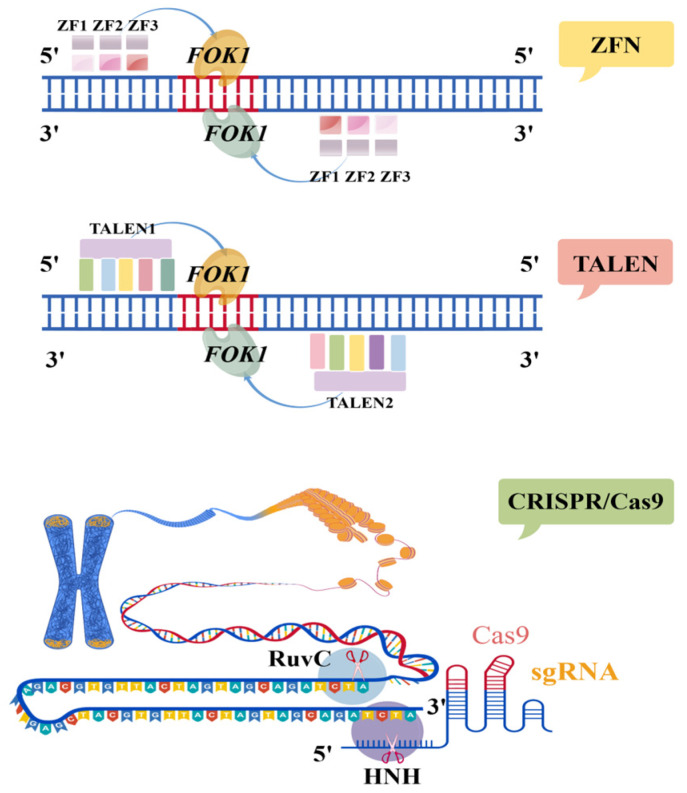
Schematic illustration of the mechanisms by which ZFN, TALEN, and CRISPR/Cas9 gene editing technologies induce double-strand breaks in target DNA (the letters represent bases on the double strand).

Although both TALENs and CRISPR/Cas9 systems enable precise genome editing at specific sites, they differ significantly in their molecular mechanisms. TALENs rely on protein-DNA interactions for target recognition, whereas the CRISPR/Cas9 system utilizes RNA-guided DNA targeting through complementary base pairing [99]. Table 2 summarizes the key differences among the three major gene editing technologies, and Figure 2 illustrates the molecular mechanisms by which ZFNs, TALENs, and CRISPR/Cas9 introduce double-strand breaks in DNA.

#### 2.1.3. CRISPR/Cas System

The CRISPR/Cas system was initially identified as an adaptive immune mechanism in bacteria and archaea. Their genomes contain short, regularly spaced repetitive sequences, and it is this distinct clustered repeat structure that encodes the molecular machinery capable of recognizing and neutralizing invasive viruses and plasmids. Owing to its programmable precision, the CRISPR/Cas system holds broad application potential in gene function studies, the generation of model organisms, and gene therapy for diseases such as cancer, liver disorders, and cardiovascular conditions [100]. CRISPR/Cas systems are broadly classified into two main classes, which are further subdivided into six types and 33 subtypes [101]. Class 1 (which includes types I, III, and IV) employs multi-subunit effector complexes composed of several Cas proteins. In contrast, Class 2 (encompassing types II, V, and VI) utilizes a single-protein effector module, as seen in CRISPR/Cas9, Cas12, and Cas13 systems. Despite the functional diversity across CRISPR/Cas systems, the CRISPR/Cas9 system remains the most widely adopted gene editing tool. Its popularity stems from its early discovery, structural simplicity, ease of engineering, technical maturity, and high editing efficiency [102,103]. The flexibility in guide RNA design and high specificity have enabled efficient and targeted editing of specific genomic sequences across a wide range of organisms [104].

#### 2.1.4. Examples of Gene Editing Applications in Industrial Microorganisms

In recent years, gene editing technologies have achieved significant advances in crop breeding, metabolic engineering, and biomedicine. In crop breeding, Zou et al. [105] innovatively employed Cas9 in place of nCas9 to develop a Cas9-mediated Prime Editing (Cas9-PE) system. This system not only enables precise genome editing but also facilitates site-directed random mutagenesis, offering a versatile and efficient tool for multiplex gene editing in molecular design breeding and substantially accelerating the development of improved crop varieties. In the field of environmental and metabolic engineering, Zhuang et al. [106] expanded the substrate range of the chloroaniline-degrading plasmid pDCA-1 through genetic redesign, enhancing its compatibility across diverse bacterial strains. This approach provides a novel strategy for genetic bioaugmentation in bioremediation. Wang et al. [107] constructed a *Saccharomyces cerevisiae* surface display system comprising the display vector pYS and the expression vector pYE, which enabled the screening and production of single-chain variable fragment (scFv) antibodies with neutralizing activity against HIV-1. This platform offers a promising new route for antiviral drug discovery. Asareh et al. [108] utilized a *Pichia pastoris* expression system to produce the therapeutic antibody ranibizumab. By separately expressing its light and heavy chains and confirming their association using chimeric enhanced green fluorescent protein (eGFP), they demonstrated the feasibility of *P. pastoris* as a eukaryotic host for Fab fragment production. In a study on metabolic pathway engineering, Han et al. [109] applied SpyTag/SpyCatcher protein scaffolding technology to co-localize key methanol metabolic enzymes—alcohol oxidase AOX1 and dihydroxyacetone synthase DAS1—in *Pichia pastoris*. This enzyme assembly significantly enhanced cellular efficiency: the recombinant strain showed a 2.3-fold increase in biomass and a 4.51-fold increase in EGFP fluorescence intensity per OD600 unit compared to the control strain, illustrating a powerful new approach for optimizing metabolic pathways.

### 2.2. Metabolic Engineering

Metabolic engineering is an emerging interdisciplinary field that enables the efficient biosynthesis of valuable molecules—such as chemicals, fuels, materials, and proteins—through the rational design and modification of microbial cell factories. It has demonstrated broad applicability across multiple domains: in basic biological research, it supports studies in bioremediation and signal transduction [110]; in medicine, it offers innovative technological platforms for gene therapy and pharmaceutical development. The typical workflow of metabolic engineering begins with the selection of a target product with high market demand and biological potential, followed by the choice of a microbial host that can be engineered to achieve high titers, rates, and yields (TRY) of the desired compound [111]. This process is iteratively refined through the design-build-test-learn (DBTL) cycle, which integrates natural or artificial biosynthetic pathways and optimizes metabolic flux using systems and synthetic biology tools.

However, conventional metabolic engineering approaches are often limited by being time-consuming and labor-intensive. To overcome these challenges, researchers have developed computational tools such as metabolic flux analysis (MFA), genome-scale metabolic models (GEMs), and algorithms like OptKnock, which can predict genetic modifications to enhance product synthesis [112]. Recent advancements have further propelled the field: novel technologies including protein engineering, CRISPR/Cas9-based genome editing, dynamic regulation strategies, and cell-free metabolic engineering have significantly improved the efficiency and precision of pathway optimization. Moreover, the scope of host organisms has expanded from conventional model systems to non-model species. The integration of machine learning techniques is also opening new avenues for intelligent design and optimization of metabolic networks [113]. These developments not only drive innovation within metabolic engineering itself but also provide a robust foundation for the sustainable advancement of the biomanufacturing industry.

#### 2.2.1. Optimization and Reconstruction of Metabolic Pathways

Optimization and reconstruction of metabolic pathways lie at the heart of metabolic engineering, with the goal of achieving high-efficiency biosynthesis of target products through systematic and rational modifications. This process entails multi-level and multi-dimensional strategic interventions: At the molecular level, metabolic flux is optimized by modulating the expression and catalytic activity of key enzymes; At the pathway level, metabolic networks are redesigned through the introduction of exogenous biosynthetic pathway or the simplification of native pathways; At the systemic level, cofactor engineering is applied to balance redox states and enhance energy utilization efficiency [114,115,116,117]. With advanced tools such as CRISPR-based genome editing for precise control of enzyme expression, dynamic regulation systems for real-time metabolic adjustment, and computer-aided design for predicting optimal pathway configurations, researchers can now engineer metabolic networks with unprecedented accuracy and efficiency.

##### Expression Regulation of Key Enzymes

Expression regulation of key enzymes is an important strategy for the optimization of metabolic pathways, among which overexpression technology has been widely used because of its easy operation and remarkable effect. Overexpression technology is widely used because of its ease of operation and remarkable effect. This strategy is mainly used to increase the expression level of rate-limiting enzymes by increasing their gene copy number or using strong promoters, so as to break through the metabolic bottleneck. As the enzyme with the lowest catalytic rate in the metabolic pathway, the activity of the rate-limiting enzyme directly affects the efficiency of the whole metabolic pathway.

**Figure 3:**
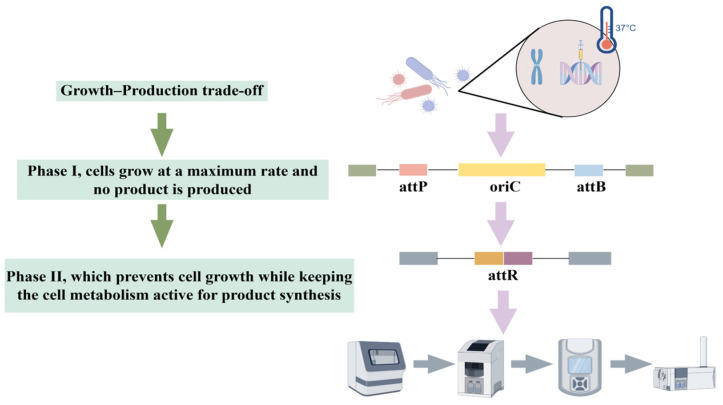
Growth-production trade-offs through modification of *E. coli*.

Studies have shown that this strategy has achieved remarkable results in different metabolic systems. By overexpressing desaturase genes *de1* and *de2*, Xu et al. [118] not only significantly enhanced the total fatty acid content of *E. coli*, but also optimized the fatty acid composition, promoted the conversion of octadecanoic acid to oleic acid, and increased the proportion of monounsaturated fatty acids. On the other hand, Zhang et al. [119] innovatively adopted a combinatorial regulatory strategy to finely regulate the expression of LysCr and Homr, the key enzymes of the L-homoserine synthesis pathway, in *Corynebacterium glutamicum* by using promoters of different strengths and RBS elements, and successfully constructed an engineered strain that synthesized L-homoserine efficiently, with a yield of 12.7 g/L, which is 3.6-fold higher than that of the wild-type strain. These studies not only confirmed the effectiveness of the key enzyme expression regulation strategy, but also demonstrated the great potential of metabolic pathway optimization through systematic engineering modification. Figure 3 depicts a novel growth switch developed by Marje Kasari et al. [120], based on the permanent removal of the replication origin (oriC) from the *E. coli* chromosome. Without oriC, cells cannot initiate a new round of replication, causing them to cease growth while metabolism remains active. Their system relies on the serine recombinase from phage phiC31, whose expression is controlled by the temperature-sensitive cI857 repressor from phage lambda. Report protein expression persists in transformed cells after growth arrest, resulting in protein levels five times higher than in non-transformed cells.

##### Metabolic Flux Analysis

Metabolic flux analysis (MFA) serves as a fundamental research tool in metabolic engineering, enabling the quantitative resolution of material flows within metabolic networks through the integration of metabolomics and isotope labeling techniques. This approach allows for the precise identification of rate-limiting steps and bottlenecks in metabolic pathways, thereby providing a robust theoretical foundation for the targeted optimization of metabolic networks.

The study of Wang et al. [121] fully demonstrated the application value of metabolic flux analysis in natural product synthesis: by molecularly modifying patchouli alcohol synthase (PTS) to improve its catalytic activity, and combining with the systematic analysis of glucose metabolism regulation mechanism, a pyruvate-sensing glucose metabolism regulation system was successfully constructed, which finally realized the efficient biosynthesis of patchouli alcohol and τ-dusonol. In the metabolic network regulation strategy, nodal regulation is an important method to optimize the allocation of cellular resources by regulating the flux distribution of key metabolic nodes. A study by Li et al. [122] revealed the profound influence of environmental factors on metabolic fluxes: it was found that inter-root CO_2_ concentration significantly affected the allocation pattern of carbon sources in melon seedlings, and long-term high CO_2_ treatment not only suppressed the accumulation of tricarboxylic acid cycle intermediate metabolites, but also down-regulated related metabolic enzyme activities and gene expression levels. This finding provides new insights for understanding the mechanism of environment-metabolism interactions and an important theoretical reference for crop metabolic engineering. Wang et al. [123] employed metabolic engineering to modify *Escherichia coli*, combined with optimized culture media and fermentation parameters, achieving an L-arginine yield of 89.7 g·L^−1^, a sugar-to-acid conversion rate of 0.377 g·g^−1^, and a production rate of 1.495 g·L^−1^·h^−1^. This demonstrates the immense potential of metabolic flux analysis in amino acid production. In Liu’s et al. [124] study, transcriptomics, ^13^C metabolic flux, and quantitative metabolomics were employed to systematically elucidate the effects of *E. coli pgi* and *edd* gene knockouts on cytidine synthesis, confirming the critical role of gene knockout strategies in optimizing carbon flux allocation and enhancing target product synthesis. Based on metabolic flux analysis, metabolic control analysis, and metabolomics results, Yang Qiang et al. [125] found that adding glycine, manganese sulfate, sodium nitrate, and other substances all contributed to increased L-threonine yield. Notably, adding inositol (0.1 g·L^−1^) and sodium glutamate (5 g·L^−1^) increased L-threonine yield by 1.86–4.9-fold compared to the control. This suggests that the combination of metabolic flux analysis and nodal regulation strategy can not only deeply analyze the regulation mechanism of complex metabolic networks, but also provide precise optimized targets for metabolic engineering modification, which can significantly improve the synthesis efficiency of target products.

##### Exogenous Pathway Introduction

Exogenous pathway introduction is an important strategy of metabolic engineering, which mainly includes two levels: gene introduction is to construct new metabolic pathways by introducing exogenous genes into the host cells. Pathway integration involves systematically incorporating multiple exogenous metabolic pathways into the host, forming complex metabolic networks. This strategy demonstrates significant advantages in enhancing the synthesis capacity of target products and expanding the metabolic functions of the host.

**Figure 4:**
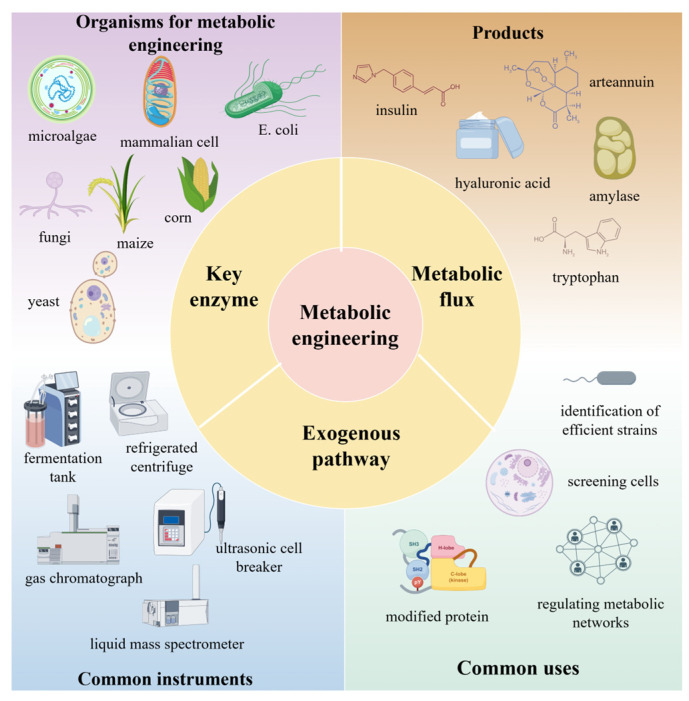
Optimization and reconstruction of metabolic pathways.

Yu et al. [126] systematically regulated the EMP pathway, PPP and TCA cycles through the introduction of exogenous sodium fumarate (SF), which not only increased the substrate level and reduced the energy consumption, but also promoted the synthesis of the major fatty acids of astaxanthin esters, and thus significantly enhanced the accumulation efficiency of astaxanthin under nitrogen-deficient conditions. This study not only elucidated the key role of the respiratory metabolic pathway in astaxanthin synthesis, but also provided a new technological idea for the efficient biosynthesis of carotenoids. The study by Jiao Feng et al. [127] demonstrated the potential application of exogenous pathway import in bioelectrochemical systems. An engineered strain with enhanced electrochemical activity was successfully constructed by introducing the Mtr pathway (The Mtr pathway is one of the most extensively studied electron transfer pathways and has been successfully engineered in *Escherichia coli* [128]) of *Shewanella oneidensis* into *E. coli*. The strain was able to utilize exogenous electrons to modulate the metabolite profile and promote the biosynthesis of succinate, which provided an important theoretical basis and technological support for the improvement of electron transfer efficiency and the development of novel bioelectrochemical systems. Exogenous pathway introduction will become more precise and efficient, opening new research directions and application fields for metabolic engineering. Figure 4 summarizes the main components of metabolic engineering, including commonly used engineered organisms (e.g., microalgae, *E. coli*, fungi), key tools (e.g., fermenters, mass spectrometers), core concepts (e.g., metabolic flux, exogenous pathways), and target products (e.g., insulin, hyaluronic acid).

### 2.3. High-Throughput Screening and Automation Platform

As a pivotal tool in modern biotechnology, high-throughput screening (HTS) technology enables the rapid detection and functional analysis of thousands to millions of samples through automated processes. It plays a critical role in diverse fields such as drug discovery and development, enzyme engineering, protein engineering, metabolic engineering, synthetic biology, and gene function research. With ongoing technological innovations, a variety of efficient and novel HTS platforms have been developed, including microtiter plate-based screening, fluorescence-activated cell sorting (FACS), biosensor-assisted screening, droplet microfluidic systems, and model organism-based screening platforms [129].

Wu et al. [130] developed a microtiter plate-based toxicity screening platform utilizing *Caenorhabditis elegans*, which enables remote operation, real-time monitoring, and automated phenotypic analysis via a web-connected “Lab on Web” system. This platform not only facilitates comprehensive assessment of cadmium-induced effects—including lifespan reduction, neurotoxicity, developmental impairment, and GST-4 expression—but also significantly shortens the toxicity testing cycle, offering an efficient and precise technical approach for environmental toxicology research. Looking ahead, the advancement of high-throughput screening technology will focus on several key directions: enhancing the scalability and reproducibility of screening outcomes, developing modular integrated systems with expanded functionality, establishing standardized software control frameworks, and promoting deeper integration of historical data with intelligent decision-making algorithms within automated platforms [131].

### 2.4. Synthetic Genomics

Innovations in next-generation sequencing (NGS), particularly the advancement of whole-genome sequencing (WGS), have significantly accelerated the study of genotype–phenotype relationships [132]. The integration of NGS with adaptive laboratory evolution (ALE) enables systematic identification of genomic variations in high-performance evolved strains and links phenotypic improvements to specific mutation clusters [133]. Databases such as ALEdb further streamline the storage and retrieval of mutation data for subsequent hypothesis validation. Beyond whole-genome resequencing in ALE studies, NGS also supports metabolic engineering through quantitative trait locus (QTL) mapping, which has been employed to identify genomic regions associated with differential tolerance to hydrolysis products in various strains of *Saccharomyces cerevisiae*. By reintroducing beneficial alleles into laboratory strains, researchers have successfully enhanced phenotypic robustness [134].

NGS-based transcriptomic approaches, such as RNA-Seq, offer powerful means to elucidate transcriptional regulatory networks in ALE-evolved strains. Incorporating such regulatory information into metabolic models—for instance, through flux balance analysis (FBA)—markedly improves prediction accuracy. The IDREAM framework exemplifies this approach, achieving precise metabolic flux predictions in S. cerevisiae by integrating genome-scale metabolic models with transcriptome-inferred regulatory networks [135]. Nevertheless, the application of transcriptomics in metabolic engineering remains less mature compared to other omics disciplines. Deeper integration of transcriptional data with metabolic modeling holds promise for enabling novel breakthroughs in the rational design of engineered strains. These technological integrations not only advance metabolic engineering but also provide innovative methodologies for systems biology.

#### 2.4.1. Construction of Minimal Genomes

Minimal genomes, as the core chassis system of synthetic biology, provide an important platform for the construction of customized genomes and the development of novel biotechnology applications [136]. Gibson DG’s research team reported the design, synthesis, and assembly of the JCVI-syn1.0 *fungal mycoplasma* genome using only 1.08 megabases of digital genomic sequence information, transplanting it into *Mycobacterium yamabushii* recipient cells to create a novel *fungal mycobacterium* cell controlled solely by a synthetic chromosome [137]. Hutchison employed whole-genome design and complete chemical synthesis to minimize the 1079 kb synthetic genome of *Mycoplasma* JCVI-syn1.0 [138]. The first successful large-scale redesign of the *E. coli* genome using synthetic biology was carried out by a team led by George Church and Farren Isaacs, who recoded the genome to eliminate all UAG termination codons, showing resistance to phage infection [139]. In advanced biological systems, artificial chromosome technology demonstrates tremendous application potential. Mammalian artificial chromosomes can be used to develop cell-based genomic therapies for cancer or to modify animal genomes for producing humanized organs or drugs [140]. Plant artificial chromosomes could make possible improved foods or plants with new metabolic pathways. In the field of industrial microorganisms, Bu et al. [141] developed a strategy combining multiple computational methods with a site-specific recombination system to construct a genome-reduced industrial *Streptomyces chattanoogensis* host platform. This strain, *S. chattanoogensis* L321, exhibits multiple novel and outstanding properties in the heterologous expression of secondary metabolites. It not only serves as a highly efficient cell factory for producing high-value polyketide compounds but also provides robust support for upgrading microbial pharmaceutical industries and advancing drug discovery.

#### 2.4.2. Design and Assembly of Synthetic Chromosomes

The design and assembly of synthetic chromosomes represent a pivotal technology in synthetic biology, with the core objective of achieving specific biological functions or research goals through the de novo construction of artificial chromosomes. This process typically involves three key steps: first, selecting appropriate host cells (such as yeast or *E. coli*) based on research requirements; second, introducing the synthetic chromosome into the host via methods like electroporation, chemical transformation, or splicing and transfer; and finally, verifying chromosomal integrity and functionality through techniques including PCR, sequencing, and phenotypic analysis [142]. This systematic workflow provides a robust framework for advanced genome engineering.

In a notable application, Huang et al. [143] innovatively identified seven exogenous gene insertion sites in the genome of *Saccharomyces cerevisiae BY4742*. Using scarless knock-in technology, they integrated the steviol glycoside biosynthesis pathway genes into the yeast chromosome, successfully constructing an engineered strain capable of efficient steviol glycoside production. This study not only highlights the potential of synthetic chromosome technology in metabolic engineering but also offers a novel strategy for the biosynthesis of natural products. Similarly, Yang et al. [144] obtained a *Saccharomyces cerevisiae* strain, yXZX565, exhibiting a formic acid utilization phenotype through screening of strains harboring synthetic chromosomes. By knocking out the *SPT2* gene, the engineered yeast demonstrated significantly enhanced alkaline tolerance, maintained cellular viability, and improved the efficiency of formic acid-mediated CO_2_ fixation. This work provides an important theoretical and technical foundation for the development of novel biological carbon sequestration technologies.

#### 2.4.3. Synthetic Genomes in Industrial Microorganisms

Synthetic genomics has achieved remarkable breakthroughs over the past decade, with its applications expanding from viral genome construction to the complete synthesis of bacterial and eukaryotic genomes [145]. In the area of viral genome synthesis, Cello et al. [146] pioneered the assembly of the full-length cDNA of the Mahoney poliovirus strain PV1(M) using chemically synthesized oligonucleotides. This study demonstrated for the first time that infectious viral genomes could be reconstructed in vitro based solely on known nucleotide sequences, highlighting the power of synthetic genomics and providing a transformative technical platform for virological research. In microbial metabolic engineering, Huang et al. [147] significantly enhanced the biosynthesis of D-pantothenic acid through a comprehensive synthetic biology approach. Their strategy involved extending cellular lifespan, improving cofactor supply, screening and chromosomally integrating the heterologous gene *panE* (encoding ketopantothenate reductase), as well as implementing acetate recycling and reconstruction of the NOG pathway. These efforts culminated in a final D-pantothenic acid titer of 5.48 g/L, underscoring the potential of synthetic genomics in industrial microbial engineering. Regarding large-scale genome assembly, the Itaya group [148] in Japan developed an innovative in vivo assembly technique. They successfully cloned the 3.57 Mbp genome of *Synechocystis PCC6803* into four separate segments and achieved stepwise integration and assembly within the *Bacillus subtilis* genome. This groundbreaking work established the feasibility of using in vivo methods for assembling large non-synthetic genomes and opened new avenues for synthetic genomic construction.

**Figure 5:**
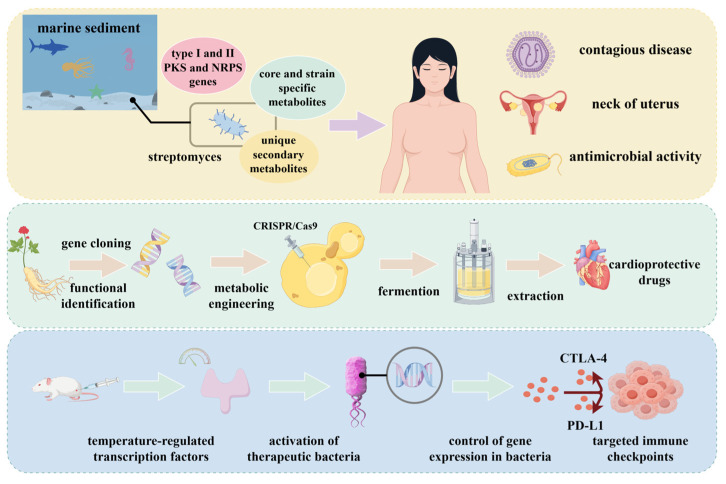
Use of synthetic biology to modify industrial microorganisms for disease therapeutic applications.

Figure 5 illustrates the application of synthetic biology in engineering industrial microorganisms for disease treatment. Edna M Sabido et al. [149] isolated *Streptomyces* strains from marine sediments. The crude extract derived from these strains inhibited the growth of ESKAPE pathogens and demonstrated antiproliferative activity against A2780 human ovarian carcinoma cells at a concentration of 2 mg/mL. Yanxin Li et al. [150] focused on ginsenosides derived from ginseng. Using CRISPR/Cas9 technology, they introduced glycosyltransferase genes into suitable microbial hosts. Batch fermentation in bioreactors yielded modified ginsenosides, and subsequent in vitro assays confirmed that these compounds protect cardiomyocytes against hypoxia/reoxygenation (H/R)-induced injury. Mohamad H. Abedi et al. [151] developed a genetic state switch activated by focused ultrasound-induced temperature changes. This system enables precise, spatiotemporal control of therapeutic gene expression in bacterial cells, offering a versatile platform for targeted antibacterial therapies across various biological and clinical contexts.

## 3. Conclusions and Outlook

As biotechnology continues to advance through innovative leaps, synthetic biology has achieved substantial progress in the optimization and construction of industrial microbial cell factories. By leveraging synthetic biology strategies, researchers can more efficiently discover and characterize biological components, as well as design and validate metabolic circuits and regulatory systems. Nevertheless, the practical application of engineered microbial cell factories still faces multiple challenges: environmental factors exert complex spatiotemporal effects on cellular energy and material metabolism, while the intricate molecular composition and regulatory networks within cells constrain the development and implementation of dynamic biological components and circuits [152]. In terms of biosafety, synthetic biology introduces several potential risks to human health, including allergic reactions, antibiotic resistance, carcinogenicity, and pathogenicity or toxicity. Environmental concerns similarly involve ecosystem disruption or resource depletion, competition with native species, horizontal gene transfer, and the potential release of pathogenic or toxic agents [153].

To overcome these constraints, future research should prioritize several key directions: First, it is essential to integrate the strengths of both model and non-model microorganisms by screening and engineering industrial strains with superior phenotypes—such as rapid growth, high substrate utilization efficiency, robust environmental tolerance, and elevated product yield—and to elucidate the associated genotypic traits and regulatory mechanisms. This will provide a theoretical foundation for the rational design of chassis cells. Genome editing technologies (e.g., CRISPR-Cas systems) and metabolic engineering tools should be further employed to optimize microbial metabolic pathways, thereby enhancing productivity and expanding product diversity. Second, building on successful biotechnological developments, synthetic biology methodologies should be refined through the creation of cost-effective high-throughput intelligent devices and the establishment of molecular genomics and phenomics research platforms. Such infrastructure will support the rapid screening of microbial strains with industrial potential and accelerate the optimization and refinement of their metabolic pathways. Finally, efforts must advance in the analysis, integration, simulation, and visualization of multi-omics and large-scale phenomic data to develop high-quality digital cell models. These models will direct genome optimization and guide the design and construction of biologically secure chassis cells. By synthesizing multidimensional data from genomics, transcriptomics, proteomics, and metabolomics, a more holistic understanding of microbial metabolic and regulatory networks can be achieved, paving the way for precise cellular factory design and optimization.

The implementation of these strategies will accelerate the engineering of industrial microbial strains, promoting their broad adoption across sectors such as chemical manufacturing, pharmaceuticals, environmental remediation, and agriculture. Moreover, progress in synthetic biology will stimulate interdisciplinary collaboration and innovation. By integrating deeply with artificial intelligence, materials science, and chemical engineering, synthetic biology is poised to enable breakthroughs in areas such as novel biomaterials, smart biosensors, and biological computing systems. For example, artificial intelligence and mathematical modeling can be employed to optimize critical parameters—including nutrient supplementation, bioreactor configuration, strain suitability, and gas transfer—to improve the growth of engineered *cyanobacteria* and thereby facilitate commercial-scale biohydrogen production. The convergence of multi-omics big data and artificial intelligence with synthetic biology can further enhance the efficiency of microbial conversion of diverse industrial waste streams into value-added products. Haotian Cui et al. developed scGPT, a foundational single-cell biology model based on a generative pre-trained transformer trained on over 33 million cells, demonstrating its ability to extract essential biological knowledge about genes and cellular functions [154]. As synthetic biology technologies continue to evolve and find new applications, industrial microbial cell factories are expected to assume an increasingly critical role in the global bioeconomy, providing innovative pathways toward green, low-carbon, and sustainable industrial development.

## Figures and Tables

**Table 1:** Examples of Industrial Microbial Applications.

Areas of Application	Examples of Industrial Microorganisms	Application	References
Chemicals and materials	*Corynebacterium glutamicum*	production of proteinogenic amino acids	[61]
	*Kappaphycus alvarezii*, *Bacillus megaterium*	production of bioplastic polyhydroxy fatty acid esters	[62]
	*Xanthomonas campestris*	production of xanthan gum for oil extraction	[63]
Drugs and vaccines	*Xanthomonas*, *Xanthomonas campestris*	production of β-glucan for biopharmaceuticals	[64]
	*Nannochloropsis*, *Saccharomyces cerevisiae*, *Sparassis crispa*, *Bacillus subtilis*	production of cyclosporin A, for immunosuppression after organ transplantation	[65]
	*Streptomyces*	production of Adriamycin for the treatment of various cancers	[66]
Food and beverage industry	*Lactic acid bacteria*	fermentation of plant-based dairy alternatives, vegetables, sweet rice wine, yogurt, cheese	[67,68,69,70,71]
	*Acetic acid bacteria*	fermented vinegar	[72]
Environmental remediation	*Thermotolerant yeast*, *Oleaginous yeast*, *Lactic acid bacteria*	production of ethanol, lipid and lactic acid from mixed agrowastes hydrolysate	[73]
	*Stenotrophomonas*, *Achromobacter*,	degradation of plastics	[74]
	*Proteobacteria*, *Actinobacteria*	degradation of oil pollutants	[75]
Agricultural	*Nitrogen-fixing bacteria*	biofertiliser	[76]
	*Filamentous fungi*	bio-pesticides	[77]
	*Soil microalgae* and *Cyanobacteria*	maintenance of soil fertility and health	[78]
Energy production	*Betaproteobacteria*	generation of oxygenated methane	[79]
	*Cyanobacteria*, *Microalgae*	biodiesel production	[80]
	*Chlorella vulgaris*, *Chlamydomonas reinhardtii*, *Chlorella fusca*	biohydrogen production	[81,82,83]
	*Saccharomyces cerevisiae*	bioethanol	[84,85]

**Table 2:** Differences between the three gene editing technologies [94,95,96,97,98,99,100,101,102,103,104].

Technology Name	ZFN	TALEN	CRISPR/Cas9
Recognition mode	Protein-DNA	Protein-DNA	RNA-DNA
Targeting element	ZF array Protein	TALE array Protein	sgRNA Protein
Cutting element	Fok1 Protein	Fok1 Protein	Cas9 Protein
Advantages	Mature platform, higher efficiency than passive homologous recombination	Simpler design than ZFN, high specificity	Precise targeting, low off-target rate, low cytotoxicity, cheap
Disadvantages	Design dependent on upstream and downstream sequences, high off-target rate, cytotoxic	Cytotoxic, cumbersome module assembly, requires extensive sequencing	Cannot be cleaved without a PAM in front of the target region, low specificity, NHEJ still produces on-target toxicity
The Potential of RNA Editing	No	No	Yes

## Data Availability

No new data were created or analyzed in this study. Data sharing is not applicable to this article.

## Abbreviations

The following abbreviations are used in this manuscript:

PLA	polylactic acid
PHA	polyhydroxyalkanoates
HR	homologous recombination
ZFNs	zinc finger nucleases
TALENs	transcription activator-like effector nucleases
CRSISPR	clustered regularly interspaced short palindromic repeats
HSCs	hematopoietic stem cells
HbF	hemoglobin
RVDs	repeat variable diresidues
Cas9-PE	Cas9-mediated Prime Editing
scFv	single-chain variable fragment
eGFP	enhanced green fluorescent protein
TRY	titers, rates, and yields
DBTL	design-build-test-learn
MFA	metabolic flux analysis
GEMs	genome-scale metabolic models
PTS	patchouli alcohol synthase
SF	sodium fumarate
HTS	high-throughput screening
FACS	fluorescence-activated cell sorting
NGS	next-generation sequencing
WGS	whole-genome sequencing
ALE	adaptive laboratory evolution
QTL	quantitative trait locus
FBA	flux balance analysis
